# Qualitative evaluation of a community health representative program on patient experiences in Navajo Nation

**DOI:** 10.1186/s12913-019-4839-x

**Published:** 2020-01-08

**Authors:** Amber Lalla, Shine Salt, Elizabeth Schrier, Christian Brown, Cameron Curley, Olivia Muskett, Mae-Gilene Begay, Lenora Shirley, Clarina Clark, Judy Singer, Sonya Shin, Adrianne Katrina Nelson

**Affiliations:** 1 University of New Mexico, 2425 Camino de Salud, Albuquerque, NM 87106 USA; 2 Division of Global Health Equity, Brigham and Women’s Hospital, 75 Francis Street, Boston, MA 02115 USA; 3 Northwestern University, 633 Clark St, Evanston, IL 60208 USA; 4 Navajo Nation Community Health Representative & Outreach Program, Navajo Nation Department of Health, Hwy 264 and St. Michael Road, St Michael, AZ 86511 USA; 5 Community Outreach and Patient Empowerment (COPE), 210 East Aztec Avenue, Gallup, NM 87301 USA; 6 Department of Global Community Health and Behavioral Sciences, Tulane School of Public Health and Tropical Medicine, 1440 Canal Street, New Orleans, LA 70112 USA

**Keywords:** American Indians, Alaskan natives, Diabetes, Patient-reported outcomes, Navajo, Qualitative, Health equity, Community health worker, Community health representative

## Abstract

**Background:**

Community Health Representatives (CHRs) overcome health disparities in Native communities by delivering home care, health education, and community health promotion. The Navajo CHR Program partners with the non-profit Community Outreach and Patient Empowerment (COPE), to provide home-based outreach to Navajo clients living with diabetes. COPE has created an intervention (COPE intervention) focusing on multiple levels of improved care including trainings for CHRs on Motivational Interviewing and providing CHRs with culturally-appropriate education materials. The objective of this research is to understand the participant perspective of the CHR-COPE collaborative outreach through exploring patient-reported outcomes (PROs) of clients who consent to receiving the COPE intervention (COPE clients) using a qualitative methods evaluation.

**Methods:**

Seven COPE clients were selected to participate in semi-structured interviews one year after finishing COPE to explore their perspective and experiences. Qualitative interviews were recorded, transcribed, and coded to identify themes.

**Results:**

Clients revealed that health education delivered by CHRs facilitated lifestyle changes by helping them understand key health indicators and setting achievable goals through the use of accessible material and encouragement. Clients felt comfortable with CHRs who respected traditional practices and made regular visits. Clients also appreciated when CHRs educated their family members, who in turn were better able to support the client in their health management. Finally, CHRs who implemented the COPE intervention helped patients who were unable to regularly see a primary care doctor for critical care and support in their disease management.

**Conclusion:**

The COPE-CHR collaboration facilitated trusting client-CHR relationships and allowed clients to better understand their diagnoses. Further investment in materials that respect traditional practices and aim to educate clients’ families may foster these relationships and improve health outcomes.

**Trial registration:**

clinicaltrials.gov: NCT03326206. Registered 9/26/2017 (retrospectively registered).

## Background

According to the most recent Navajo Mortality Report, cardiovascular disease and diabetes are among the most prominent causes of morbidity and mortality on Navajo Nation, accounting for the 3rd and 4th leading causes of death respectively [1]. Between 2010 and 2013, 25,000 Navajo tribal members were estimated to be diabetic and 75,000 more to be pre-diabetic [2]. The burden of chronic disease on Navajo Nation stems in part from historically unjust policies toward Native communities across the United States. Modern barriers to accessing care, including long distances to health facilities, unpaved roads, and little healthcare funding resulting in limited resources and coordination of care further exacerbate the problem [3]. However, since 1968 the Navajo Nation Community Health Representative (CHR) Program, a community outreach and health promotion program has successfully helped to mitigate these barriers. Carried out by trained tribal or community members, the Navajo Nation CHR Program addresses social and health access issues in a culturally-appropriate way [4, 5].

In evaluating impacts of such community-based programs, it is widely agreed among clinicians and researchers that units of measurement traditionally associated with clinical and effectiveness trials, such as routinely collected health indicators and health utilization outcomes alone fall short in capturing the full picture of patient experience [6–8]. Increasingly, Patient Reported Outcomes (PROs) have emerged as a valuable measure to supplement clinical outcomes in the evaluation of health services and interventions [9–12]. According to the Patient-Centered Outcomes Research Institute (PCORI), PROs, “help people and their caregivers communicate and make informed health care decisions, allowing their voices to be heard in assessing the value of health care options” [13, 14]. For researchers, PROs can provide a deeper sense of the patient experience, glean insight into why a program may or may not work, and place the patient in a role of partnership with clinicians in care management. The emerging use of PROs has showcased the importance of the patient perspective in ensuring high quality and appropriate interventions for the priority population [15].

The evaluation of PROs typically involves a questionnaire, qualitative interview, or focus group capturing data that is either subjective or not traditionally captured in electronic health systems. Examples of such data include information about trust and comfort with care, experience of recovery processes, quality of life, perceived health status, understanding of clinical indicators, and behavior patterns. Most notably, it allows patients to direct the research toward matters that are most important to them.

PROs could be particularly useful for research in Native communities, where interpretation of health behaviors by clinical staff and researchers unfamiliar with the local context may be erroneous, and patients may access care intermittently, either due to distance or mistrust of non-native health providers. To our knowledge, there are no research articles published about healthcare in tribal regions explicitly using PROs. In this paper, we seek to use PRO’s to understand the patient experience in the community-based lifestyle intervention referred to as Community Outreach and Patient Empowerment (COPE) Intervention.

### Study setting and population

**Fig. 1:**
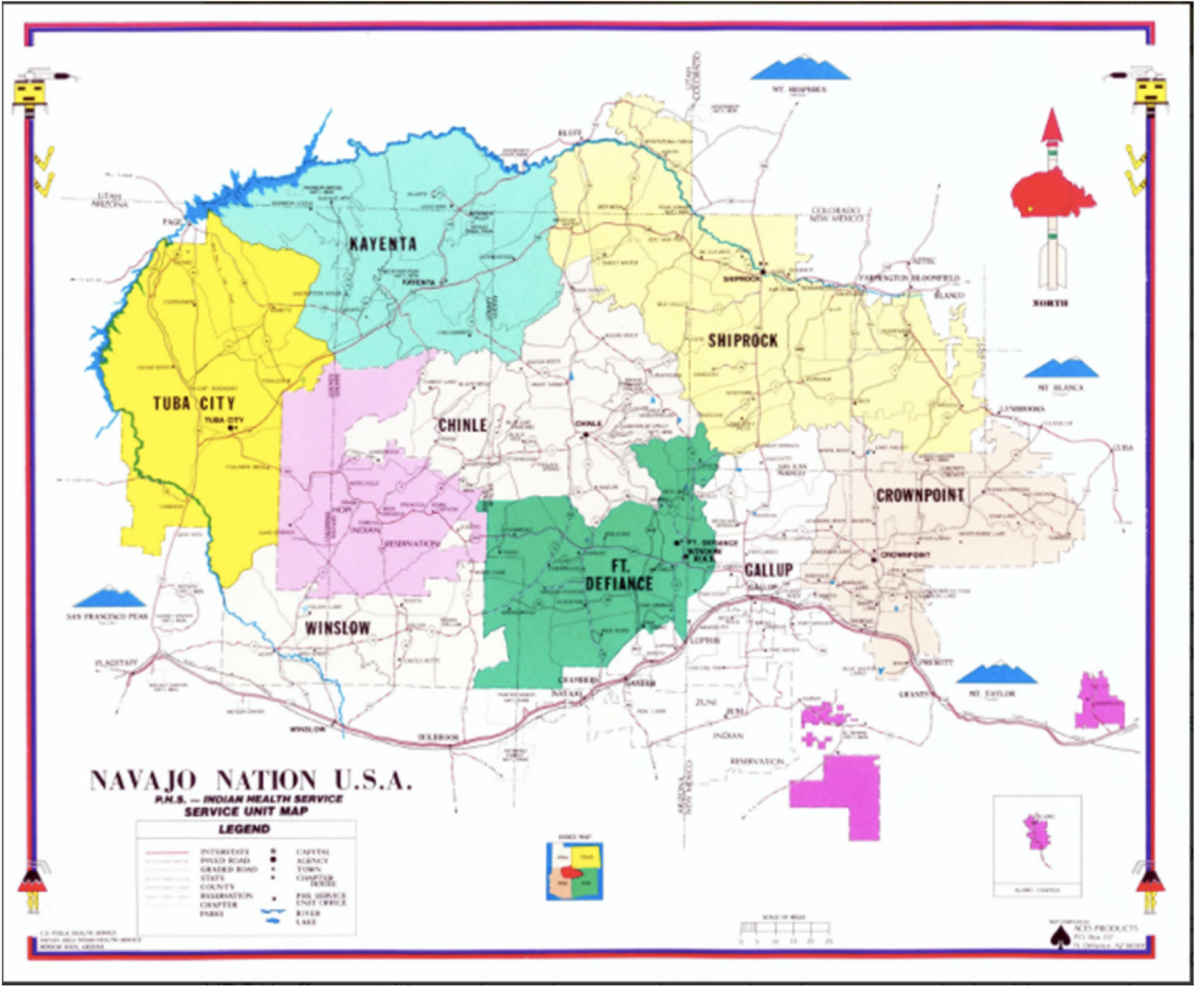
Map of Navajo Nation showing geographic Indian Health Service Unit boundaries (Image credit: https://www.ihs.gov/navajo/)[27]. This image was created by the US Public Health Service- Navajo Area Indian Health Service and is not protected under copyright. Available under license: CC BY 4.0

At roughly the size of the state of West Virginia, the Navajo Nation is the largest Native American reservation in the United States, comprising more than 27,000 miles^2^ and encompassing parts of Utah, Arizona, and New Mexico (Fig. 1) [16]. The tribe has over 300,000 enrolled members [16] and about 200,000 residing on the reservation [17]. The Nation Area Indian Health Services (IHS) runs the tribe’s four in-patient hospitals, seven outpatient health centers, and five part-time health stations and also has five additional hospitals through Public Law 93–638 contracts with tribal health institutions within eight service units (SUs). Within these SUs there are 110 geographic chapters – the most local form of government for Navajo Nation, each with a chapter meeting house where chapter members may attend meetings and vote on local issues.

#### The CHR program

The Navajo Nation CHRs work to strengthen individual and community capacity by increasing health knowledge and empowerment through a range of activities such as home visits, health education, social support, referrals to services, and advocacy. CHRs use the term “clients” to refer to patients they see in their homes, and for the purpose of this paper, “clients” will be used when referring to the “patients” assigned to CHRs. CHRs are trained in HIPAA and to deliver health assessments, including blood pressure, blood sugar, and heart rate. CHRs visit clients’ homes two to three times a month, depending on the condition of the client, and up to eight clients per day. The Navajo Nation CHR Program works across all eight SUs, which are each assigned between six and 18 CHRs. Currently, there are 85 active CHRs on Navajo Nation.

Despite the long-standing and positive presence of CHRs in the community, efforts to build strong communication and collaboration between CHRs and clinical teams have been limited. This is in part due to different tribal institutions overseeing clinical versus community-based programs (e.g. Indian Health Services (IHS) and the Navajo tribal-owned CHR program). In order to further integrate CHRs into the Navajo healthcare system, in 2009, the Navajo Nation CHR program, Brigham and Women’s Hospital, Partners in Health, and the Navajo Area IHS began a formal collaboration called COPE. COPE aims to strengthen the CHR referral system within clinics, support CHR client accompaniment through education materials production and trainings, and facilitate communication between providers and CHRs. As part of the COPE/CHR collaboration, clients with uncontrolled chronic diseases (such as diabetes, heart disease, high blood pressure, etc.) are prioritized for regular home visits from CHRs and health education in at least 10 of 36 topics. Clients choose the health topics of their choice and often receive more than 10 if time allows. To date, approximately 590 patients have been enrolled in COPE. Strategies for increased communication to date have included creating access for CHRs to Electronic Health Records, case management rounds, volunteer health providers leading CHR training on health topics, and orientation to new healthcare providers about the role of CHRs.

## Methods

### The COPE intervention

The “COPE Intervention” is comprised of three inter-related strategies designed to strengthen existing community outreach and linkage to clinic-based care: 1) Referrals to CHRs: COPE staff have worked with healthcare providers to raise awareness of the services provided by CHRs and to strengthen referral systems that allow providers to refer patients to the program and share relevant clinical data such as medications, pending appointments and laboratory tests with the CHR. 2) Providing CHR trainings in community-based patient education and coaching to promote behavior change. COPE sought to strengthen support of CHRs in their work with their clients by providing training in evidence-based behavior change strategies, including Motivational Interviewing (MI), patient-identified SMART goals (i.e. Specific, Measurable, Attainable, Relevant, Time bound), and structured health education materials in the form of flipcharts. Guided by CHRs, COPE developed each flipchart to cover a specific health topic (e.g. cholesterol, diabetes foot care, physical activity) based on clinical guidelines drawing from culturally-informed materials and feedback from local stakeholders. Each topic, or module, follows the format of a brief counseling intervention known as the 5A’s (Ask, Assess, Advise, Assist, Arrange follow-up). The 5 A’s have been demonstrated to be effective in promoting behavior change as well as chronic disease management [18]. 3) Increasing community-clinical linkages in coordination of care: COPE has worked with both CHRs and providers to increase bi-directional communication about client issues, such as medication changes and side effects, abnormal symptoms or vital signs, and changes in social support. COPE also provided CHR clients with low-cost items designed to facilitate client-identified behavior changes, for example, visual material for charting clinical indicators and setting goals, measuring cups, exercise bands, and portion plates (Table 1). Participants in the COPE intervention stay for at least one year and are invited to stay on as they feel they need to afterward.

**Table 1:** Community Outreach and Patient Empowerment (COPE) Intervention Components

Program Components	Before COPE collaboration	Introduced by COPE collaboration
CHR Training	Community Health Representatives (CHRs) receive training on health topics when available.	Monthly training sessions to CHRs on health topics taught by local providers to build CHR-provider relationship.
	CHRs do not receive training on motivational interviewing, self-care, goal setting.	CHRs receive training on motivational interviewing, self-care, goal setting delivered by Navajo-speaking trainers.
	No competency assessments of CHR or trainer knowledge / proficiency.	Competency assessments administered at each training to assess CHR and trainer knowledge / proficiency.
	CHR supervisors receive training when available.	CHR supervisors receive monthly trainings in team building, supervision and leadership, quality improvement, and wellness / self-care.
Patient outreach	Home visits by CHRs without established frequency.	“COPE clients” receive home visits at least monthly and tracked as high-risk client.
	Each CHR prepares his/her own health education materials resulting in inconsistent health coaching.	CHRs deliver standardized coaching materials that have been vetted by local providers and ensure goal setting at each session
	Vital signs monitored inconsistently, CHRs lack oximeters, multiple size blood pressure cuffs, or glucometer training / supplies.	Vital signs monitored; all CHRs equipped with oximeters, multiple size blood pressure cuffs, glucometer training / supplies.
Community-clinical linkages	CHRs work with Public Health Nurses to evaluate clients together and establish care plans; however, CHRs rarely coordinate care with other healthcare providers.	Increased bi-directional communication and care coordination through planning conjunct meetings, orientation of new clinical staff, provider-led CHR trainings, joint home visits, and conjunct case management.
	No access to Electronic Health Records (EHR) for CHRs.	CHRs are able to gain access to EHR to document home visits and obtain client information.
	Patients rarely referred by providers to CHRs; primarily identified by CHRs themselves.	COPE helped to increase the awareness of the CHR program with presentations in hospitals. Referral system established and increased referrals by providers to CHR Program.

This study is part of a larger Patient-Centered Outcomes Research Institute (PCORI)-funded trial, which aims to understand how COPE is positioned within the Navajo health ecosystem and how the “COPE intervention” impacts patients’ health outcomes and sense of empowerment. Quantitative findings from this study demonstrated that over a two year period, participants enrolled in the COPE intervention (*n* = 173) significantly decreased their glycosylated hemoglobin and low density lipoprotein levels by .63% and 4.6 mg/dL, respectively when compared with non-intervention patients (*n* = 2885) [19].

### Aims and objectives

While these previous findings demonstrate the success in improving clinical outcomes, the objective of this paper is to understand the patient perspective through PROs from patients receiving the COPE intervention. Specifically, we will describe PROs one-year post enrollment with the COPE/CHR collaboration in order to:

- Better understand PROs for COPE participants
- Better understand individual experiences with COPE
- Identify keys to the program’s success

#### Study design

This is a qualitative study using a descriptive approach [20]. Utilizing a semi-structured interview guide, we conducted interviews from March 2016 to January 2017 with seven COPE participants enrolled for approximately one year to explore how patients achieved changes in their PROs. All participants interviewed had a history of diabetes mellitus (Table 2).

**Table 2:** Demographic and clinical data (*n* = 7)

(n, if not 7)	Mean or n(%)
Age	60.14
Male	6 (85.7)
BMI (6)^*^	36.03
Electricity in home (5)	3 (60)
Plumbing in home (5)	3 (60)
Heating type (4)	
Pellet	1 (25)
Wood	3 (75)

*BMI of 18.5–24.9 = normal; 25.0–29.9 = overweight; 30.0–34.9 = Class I obesity; 35.0–39.9 = Class 2 obesity, 40.0 + = Class 3 obesity

Upon encouragement from the CHRs the study staff decided to use qualitative interviews to understand PROs pertaining to participants’ health, sense of control and coping. Questions were focused on exploring personal experiences over the past year in managing their health as well as experiences with their CHR and the COPE Intervention’s materials and training, including flipcharts and goal-setting. The study staff developed the semi-structured interview guide to include two sections: participant experiences in their management and clinical outcomes, and experiences in clients’ relationship with their CHR (Additional file 1).

#### Sampling and recruitment

Study participants were recruited through convenience sampling. CHRs in all service units were asked to identify COPE participants for qualitative interviews. Four of the eight SUs (Chinle, Fort Defiance, Gallup, and Crownpoint) were represented. CHRs introduced clients to interviewers from the study team, who reviewed the study procedure, obtained informed consent, and scheduled and conducted interviews. Interviews were scheduled one year after the participant was first enrolled in COPE, with a window of 3 months before or after their first anniversary. The study team conducted interviews at the location of the participant’s choice, typically in their homes, and lasted 45 to 60 min. At least two members of the study team were present during interviews (one Navajo native speaker- CClark and the other alternating between one Navajo (SS or CB) and one Non-Navajo study team member- AL or AKN). Most interviews alternated between both Navajo and English.

The study staff identified 13 clients being visited by seven CHRs based on accessibility (having participated in a previous COPE survey) and time in the COPE intervention (one year, with a window of three months). CHRs were used to access clients in order to maintain transparency and honor COPE’s collaboration with the CHR program. Ultimately, five CHRs responded and seven clients consented to an interview. The other two CHRs were unresponsive after multiple attempts to contact them. Three of the eight service units (Fort Defiance, Gallup, and Crownpoint) were represented in individual interviews. CHRs were not present during the qualitative interviews. Participants were given an incentive of a small gift card ($20) to take part in the interview.

Interviews were audio-recorded and transferred to a secure device upon completion. Each recording was de-identified and transcribed by a trained Navajo-speaking member of the study team (CClark). Parts in Navajo were simultaneously translated into English. In addition to the interview transcriptions, each interviewer completed a debrief form reflecting on their recollections and impressions of their interviews (Additional file 2). Participant families were encouraged to participate, upon consent by the participant. Interviews with family members present are indicated as (participant name and family member). Participant names were changed to protect their identity. Quotes that were translated are indicated here by “[Speaks in Navajo]” before the sentence.

#### Qualitative analysis

The study team used reflexive thematic analysis approach as developed by Braun and Clarke to analyze data [21]. Using inductive reasoning, a codebook was developed and approved by all members of the study team. Content itself drove the identification of emergent themes and the organization of codes into themes. The study team (AKN, SS, CB, CClark, CCurley, ES and AL) double-coded interview transcriptions and debrief forms in Dedoose version 8.2.14. Team members collectively summarized excerpts for each code into a short paragraph. Finally, paragraphs were combined to form a narrative using a semantic approach intended to report the reality as depicted in the data.

#### Community advisory groups approval

Our qualitative interview protocols were developed and interpreted collaboratively with two stakeholder groups. The COPE Advisory Group (CAG) established in 2012 is comprised of local physicians, nurses, program leaders, information technology specialists, Navajo Nation Department of Health program directors, and CHR supervisors, and the Community Health Advisory Panel (CHAP), established in 2013, which includes COPE participants, their relatives, and CHRs. CHAP clarified the wording of questions in the qualitative interview guide to limit confusion and guided culturally appropriate items in September 2015. Questions about traditional food consumption were added by both CAG and CHAP groups in January 2016. Both groups interpreted preliminary results during at least two time-points during analysis (March and May 2017). Each group meets three to four times a year to provide oversight and input for COPE’s services and research.

## Results

The PROs described by participants were organized into five themes including: how CHR education and coaching (1) improved health literacy and (2) provided tools for self-efficacy and control of their lifestyle. Then, 3) recovery is life balance and (4) family caretaking and support. Finally, 5) CHR bonding describes how CHRs delivering COPE provided clients with comfort and support not experienced with their primary care provider. All five themes included common threads of understanding and regaining control over health issues and provided insight on how COPE’s materials and trainings impacted their experiences with their CHRs. These results also highlighted ways that the CHR-COPE and other CHR support programs could improve client experience in the future.

### Improved health literacy

Overall, participants believed they had better understanding of their clinical numbers after the study year. Interviews demonstrated that participants cited patient education provided by their CHR supplemented by COPE materials as the cause of this increase. Participants explained not fully understanding what their numbers represented, but could interpret them as being in healthy or unhealthy ranges, which allowed them to take a meaningful role in their illness management, by checking, tracking, and charting their numbers.

*Yeah, Alc every 3 months, what your average is, and the last time mine was 8.5…It was way higher before so, I’ve been improving… I check my blood all the time and, uh, pretty good. Down to 98, 96, and 120, 130 in the morning. (Robert)*

For people without diabetes, the normal range of A1c hemoglobin is between 4 and 5.6%. Diabetes is defined by having a A1c hemoglobin of over 6.5% [22].

When asked how well they understood their numbers, about half of participants admitted to not fully understanding what the numbers represented. Rather, they focused on whether numbers were in ‘healthy’ or ‘unhealthy’ ranges.

*|:Do you feel you understand what Alc and blood pressure kind of means and what they stand for?:|* *To be honest, I didn’t even know about that. To me it’s just a number. If it’s too high it’s not good and too low it’s good. That’s just the way I think about it. It’s like no good and good. (Jonathan)*

Participants appreciated the CHRs’ use of visual materials to help them understand and interpret these ranges, particularly for measures that participants took by themselves on a regular basis, such as blood sugar or blood pressure. A few participants specifically cited the visuals on the flipcharts as helpful in aiding their understanding of the material.

*When she has time, she comes over here. Brings books and we read together. Then she explains it to me…She brought pictures and I understood from those pictures, including the flipcharts. [Navajo; I like them coming over to visit] I wish they could come all the time, like mostly everyday (Laughs). (Marie)*

*She’ll bring out little pamphlets like and then he’ll understand what it’s for, like for his feet and then umm… Then, what else? Like you said his A1c, he has it up on the wall so he knows, and then his blood pressure- um his sugar level, she marks it where it’s good and where it’s not good. So, he has it up on his wall so he knows that… (Shuman + family member)*

### Tools for self-efficacy and control

Almost all participants explained that being able to understand their clinical numbers using visual materials helped them feel more in control of their health by making concrete changes to their lifestyle such as responding to their own symptoms though exercise or diet.

*She kind of really woke me up. You know, to things, my foot, my A1c, good to look at it.* ***|:Okay:|*** *Much more better. It. and aah, took it myself. And people like that are angels to me and other people as well. (Sheldon)*

One participant demonstrated using his newly acquired knowledge of high and low blood pressure numbers to inform when he needed to take walks or rest.

*He knows his blood pressure. He knows if it’s high. Like I’ll check his blood pressure and if it’s like 145, he’s like “Oh, it’s high.” Sometimes I have to like, he doesn’t really know, but I let him know that the top one, if it’s high and it’s not good. The bottom is always good. It’s always the same. But the top is… when I tell him, it’s high. Then he’ll be like “Okay I need to go for a walk” or “I need to lay down.” (Shuman + family member)*

Participants described CHR coaching as contributing to their incorporation of more healthy behaviors. In our interviews, participants explained that setting and achieving small lifestyle goals, referring to the SMART goal portion of MI, not only improved their physical health, but also contributed to their sense of empowerment.

|:*How has your CHR helped you make changes in your lifestyle?:|* *[Navajo; Yes] Instead of staying on the couch, do something around the house, she said. At least take a walk to the highway and come back or walk around the house, she said. I do that. (Marie)*

Many interviewees spoke most positively of their changes in their eating habits. About half of participants described making an effort to limit portion sizes and eat healthier.

*Like last night…, my wife did a good job of cooking, and aah, I just had one serving. Good stuff like that, I usually have two. I really had to think about 'Okay, one serving’s good enough.' (Laughs) (Robert)*

*I totally got away from sodas. I don’t drink sodas no more. Bread, I don’t eat no more. Just water, constantly, every day, I drink a lot of water. Junk food I don’t eat anymore. Even when we eat out, my family, I just get a salad while they eat their burgers. It kind of changed my mind around it. It doesn’t bother me, like if she was eating a triple-double burger, it doesn’t bother me anymore. It used to. I used to think, “Ughhh, I want one too” but I had to change my mind I said. So that part, I had to do that myself.* *|:And what made you decide to make that decision to change?:|* *Well I just woke up one day and said this life isn’t for me. Just sitting on the couch doing nothing. Just getting bitter by the day. Yeah, that’s just what I thought. (Jonathan)*

Our findings suggest that a combination of health education and MI techniques, including the setting of SMART goals, provided participants with an understanding of simple healthy behaviors that can improve their sense of control of their health. When these preliminary results were presented to COPE’s CAG, they confirmed the study team’s interpretation that sufficient understanding of how to interpret clinical markers as within ‘healthy’ or ‘unhealthy’ ranges allowed COPE participants to feel more in control of their health.

Almost all participants discussed gaining knowledge from their CHRs through health education. These individuals recalled CHRs using educational materials such as COPE flipcharts, incentives, and brochures and found the materials helpful in allowing them to understand their diagnoses.

*[Navajo; Yes, she explains it nicely. She says] She explains to me real good on how to eat. What to eat and all this, not too much, just a little portion. (Marie)*

*They told me, when he’s going to drink juice, this is how much he’s supposed to drink. Apple juice, orange juice, pineapple juice. Don’t drink a whole lot… Yeah, he got a little tray too. Whatever he’s going to eat, he’s supposed to have the tablespoon full on each of them. Meat, he just has about this size. (Caregiver, David)*

Secondary to changes in diet, walking was a particularly popular form of exercise and was mentioned by almost all participants as an easy way to stay active.

*Just doing a lot of exercising and walking. I think that’s what changed the whole thing. My Alc and blood sugar all went down. I think that’s what it was, just a lot of exercising and umm, usually wake up early at 5 and go walking before I go to work. (Vincent)*

While participants spoke more about changes in their diets than exercise, they did indicate an interest in learning more on how to keep active. These results suggest that COPE participants benefited from previous health education and wished to expand their health education to other areas of improvement in their own health management.

### Recovery is life balance

Participants often spoke of their experiences in coping with their physical, mental, and spiritual health. When asked about skills they had acquired to become more capable of managing their illness, participants cited their personal, emotional, and spiritual experiences as helping them gain meaning and balance in life.

Interestingly, in our interviews, participants correlated their initial chronic disease diagnosis before being engaged with the CHR program with a distressing life change or loss. This corresponds to significant literature supporting correlations between stress and glycemic control [23].

*Sometimes I’ll feel down. I just have to keep myself busy just to forget it. Most of the time it was depression because I lost my father back in 2011. After that, because of all the depression, I started being a diabetic. My sugar level was 400. Way up there. My A1c was 135 and ever since I started going in for, my uhh, surgery… my journey, I’ve gone down all the way to 6 on my Alc.* *|:Oh wow, great:|* *And then my umm… what do you call it… that body… body… was it BLM…* *|:Oh, BMI:|* *Yeah, BMI down to 59. (Jonathan)*

*The way my health is like, umm, back in 2008, before 2008 I use to take care of my dad. Every time I took him to the hospital, they use to check my blood sugar and everything. Everything was normal until he passed on in 2008. I didn’t, no way, not lonely or anything but somehow I guess my body just felt that way. All of a sudden I had heart pain and about a month and a half after he passed on I had a slight heart attack. Then when I went to the hospital they told me my blood sugar was over 300. (Vincent)*

Therefore, recovery involved physical, emotional, and spiritual healing. Without being prompted by the interviewers, about half of people interviewed identified Navajo traditional-spiritual practices as helping them gain clarity with poor health and troubling life-changing events. About half of participants described the importance of traditional knowledge, which included listening to medicine men and elders, speaking Navajo, and paying attention to dreams. Others also used church, prayer, exercise and arts and crafts.

*[Navajo: When you speak Navajo to your relatives, they say to ask your elders when you have a question, when you are in doubt, and if you’re ever in a bind. That’s how it seems, when you’re lost. And you ask questions. And in the middle of it, you realize it. It’s like you get lost, but you realize it. I was told that you can ask them for prayers, from elders. Your strong prayers. I believe in my dreams. My dreams too, I look at that.] (Sheldon)*

*I do artwork, I do that and wood carving. I dove back into it. It kind of brought me back up. I felt better after that… I think it’s just your family, your prayers, the things that are really meaningful to you. Like for me, I do a lot of artwork. I paint. I do pottery. (Vincent)*

In an attempt to tease out concepts of illness self-management and balance with regards to COPE’s role, questions to clarify how these concepts fit into participants’ evaluation of health were worked into the qualitative interview. Participants described balance as a holistic concept that includes physical, mental/emotional, and spiritual well-being. Self-management mechanisms explicitly taught by the CHR, such as eating healthy food and exercising, were seen as activities that added to participants’ sense of balance.

*Yeah, I try to set aside a time for my prayers and then exercising more. Try to stay away from the foods that are not good for me. I try to do a lot of walking. Lately, ever since it got much more colder, I haven’t really been doing that. So I feel the way, it seems like, when I use to walk all the time I use to be energized and ready to do things. Now I kinda like, don’t feel up to it sometimes but I think that’s what mainly changed. (Vincent)*

*That's what I'm about, about my faith. I’m not to supposed to share my faith, I’m supposed to live it. I’m supposed to be healthy, be able to walk, balance, mentally, spiritually. (Robert)*

About half of participants expressed this sense of balance as something they strived for, could be obtained with effort, was clearly either present or not, and could be regained when lost.

*I was raised with the traditional people from way out there, way back and they really helped me, too, with my balance and my prayers, speaking Navajo. Surely, that really helps. [Navajo:…See uhh… see when my dad died… probably in 2000, I lost my first son on the Hopi side. It’s that guy with the football jersey. That’s when it gets you down, you try to get up to balance, but I haven’t done that.] (Sheldon)*

While participants described clear improvements in coping and health-specific behavior changes during their year in COPE, overall life balance, described by participants as broader holistic wellbeing, did not change dramatically within that same time period. While this is consistent with the ongoing, dynamic life journey that shaped their overall spiritual and holistic wellbeing, traditional-spiritual practices and the arts were important to participants and addressing them may help them achieve their larger health and wellness goals.

### Family’s participation enriched health benefits

Within the interviews, many participants mentioned receiving regular visits and support from family and friends. Among those that did receive support, family members emerged as the most important form of social support especially with health management. When asked in the interview about who they were closest with, almost all described being closest with family members and a couple identified other close friends.

*|:Who do you say is the closest to you in your life right now?:|* *I would say my family and then my aunts because we talk. Just like they say, A family that prays together stays together. That’s how I look like at it. If I need to talk about something there’s someone I can talk to.* *|:How do you support each other through that|:|* *Umm, just through words. (Jonathan)*

*You know I used to think about getting old. It seems like it was just there all the time. My birthday would come up, I use to think, I’m just getting old. That’s what use to come to my mind. I have some friends who really like my artwork, they kind of give me a boost. They say, keep it going. That’s fine work you do. Then one of those friends, she sent me a card for my birthday. Then on top it says, “You’re not getting old” and inside it said, “You’re a masterpiece in progress.” Just by reading that, it seems like it turned the whole thing around where you began to see the quality in your life. I think sometimes simple things can turn around where you’ve never thought of it. So now I want to work on that, be the masterpiece. (Vincent)*

In interviews, half of participants’ family members helped in a variety of ways such as preparing healthy meals, providing transportation, and/or helping monitor medication.

*My daughter tells me when to take the medicine and how much to take. Sometimes I don’t remember how many I’m supposed to take and when they tell me to take, I don’t remember. And then much later I’ll remember what I was doing. (Marie)*

Not only did participants describe their family’s role in their health management, family members were present in the home in almost half of the interviews, and in a couple of cases, joined in on the interview. Because family members were frequently present in participants’ homes and played an important role in their health management, CHR visits also facilitated family health education. The two family members who joined in the interview described also sitting in on CHR visits.

*|:What do you enjoy most when the CHRs are around during home visits?:|* *We like it when they tell us what to do and how to take care of himself, everything, taking medications. He’s trying his best to do what he was told. (Laughs) (David + family member)*

Overall, participants acknowledged the importance of family and friends in their ability to manage their health. Additionally, participants showed interest in more resources that better incorporate their support system in their health management. These data suggest that the COPE-CHR partnership should explore additional materials and training for clients’ family and friends in the future.

### CHR-client bonding

Almost half of participants described their relationship with their CHR as casual, friendly, or familial, which made visits enjoyable. CHR clients characterized their CHR as a confidant, who would not only listen to their perspective on their personal lives and health ailments, but would also be able to understand, provide advice, and to respond adequately to health needs. A common theme was feeling more comfortable discussing their personal lives with a CHR than the client’s provider because of this close relationship.

Most of the people interviewed mentioned a frustration with the healthcare system, highlighting turnover and lack of intimacy with their provider.

*Yeah, it’s like I’d rather talk to the CHR because they’re closer to me. And those doctors over there they constantly switch over. I’ll see this person then the next time I go there there’ll be a different one. I have to explain the same thing over and over again. So the CHR already knows… they know my problems, my journey, they know what I’m going through. Then when they see me they’ll say, ‘Hey, how are you doing?’ (Jonathan)*

The high rate of turnover of providers made it difficult for participants to build a lasting and meaningful relationship, which in turn generated disappointment.

*But I heard that, the doctor that we go see took off again and we have to go to another doctor. We had an appointment to see another doctor. I don’t know when.* *|:And that makes it a bit difficult, traveling further:|* *Yeah. We use to see one in =[Location 4 Name Omitted]= he said he was going to take a month vacation. He’s from =[Location 5 Name Omitted]=, going back over there for one month. He gave me an appointment again in two months, and here I was waiting for it and they wrote a letter saying the doctor found another job in =[Location 5 Name Omitted]=. He’s not coming back. (Laughs) (David)*

*|:Do you know the longest time you’ve had one provider? What was the longest time that that provider was your provider?:|* *Yeah. That’s the one that I was… [Navajo: Three months]* *|:Three Months is the longest:|* *Yeah. Yeah, then uhh, I don’t know. It might change. I gotta, I guess… just the way I see it, [Navajo; Are they just learning on us] I don’t know how to explain it, but they just, they just do tests on us. Then they go to another hospital. I mean, maybe just they just train- [Navajo: Practicing] Practicing on us, on us Indians. Maybe they just practicing on us. And I don’t know if they’re rotating or going onto the next hospital. (Sheldon)*

A few participants expressed shyness or discomfort with providers who they were unable to build a trusting bond with, one participant suggested that some nurses were more approachable. Of note, participants tended to view any healthcare provider in a clinical setting as a doctor, and not necessarily differentiate between physician assistants, nurses, physicians, specialists, etc.…

*|:So, do you feel comfortable talking to her or does the accent make it harder:|* *Yeah, uh, I don’t want to keep questioning her like that. She might, then she might um… take it the other way I think. So, uh, sometimes there’s some nurses that work together to them. She asked them to come in and they will explain it more. So I don’t know. (Sheldon)*

One participant expressed discomfort with doctors over-prescribing medications and occasionally questioned their judgment.

*From the doctor they’ll tell you, you need to take your medication. I tell the elders too, if you take your medications everyday go back, get refills and get medication, just take it daily. What if you’re at the normal level again and doctor just keeps telling you, take it, take it. You guys need to realize that and kind of, maybe I shouldn’t say this but I tell them, maybe go without your medication for a day and see how you feel. Cuz I don’t want you relying on the medication, just that alone. Maybe you’re well and you’re still taking medication, who knows. I just tell them that. (Vincent)*

Participants described feeling comfortable discussing their personal and social lives with their CHRs because of their community and tribal connections. Over half of CHR clients interviewed spoke about having CHRs who shared their clan, or ancestral lineage, and a few said that this was an important factor in building trust.

*I can tell her anything about my personal life. She’ll talk to me about it. I think they’re both like that because in this community, we’re all related. I think =[CHR 4 Name Omitted]= is like my grandma and =[CHR 2 Name Omitted]= is like a cousin sister to me. So if I need to talk, I would talk to them with my health.* *|:So having that clanship really helps.:|* *Yeah. Other than that, some other person I wouldn’t tell about my personal life or problems and all that.* *|:And you feel safe sharing that, like she won’t judge you or those kinds of things.:|* *Yeah, they’re both nice.* *|:Do you usually share things with them?:|* *Yeah, yes I do. I tell them about my love life. (Laughs) (Jonathan)*

*|:How was your relationship? Did it change with your CHR in the past year? Okay. You said it was his mom huh? (To daughter):|* *Clan-wise, yeah. He calls her his mom. (Shuman)*

Participants who felt comfortable with their CHRs were able to find additional emotional support and joy in their visits. One CHR client explained that simply talking to her CHR makes her feel better and motivates her in taking control of her health.

*Well, she talks about herself and I told her about myself. Then it makes me feel better when I talk myself, I do this and that. So we talk to each other, like a daughter and mother.* *|:Good:|* *But I can’t talk to my kids like that - they get pissed off, so it’s better to talk to another person. It makes you feel better. When you talk to them, they talk the same way like you do. That’s why she makes me feel better when she comes sees me… Yes. It’s a real help...She was a real help for me to get it right, do okay. Go live and look forward to another day. (Marie)*

Finally, CHRs provided instrumental support by reading their vitals, blood sugar, and blood pressure in almost all cases, picking up medication and providing equipment such as test strips in over half of the cases, or arranging or reminding them about appointments in almost half of the cases.

*I needed to see a neurologist for my, so =[CHR 4 Name Omitted]= and went and set an appointment for me in =[Location 2 Name Omitted]=. When I need some medication, they would pick it up for me. I’d just call them or for more test strips, they would get it for me. It would save me a lot of time leaving here and going there to pick it up. They’ve been helpful. (Jonathan)*

*I think- At one time, like I said I was kinda, she went and got my, my medication. She went down and drove down and got- I had a refill, had a refill done. So I could get, I had them pick it up. I asked- I was going to go in the next day but then she came and I told her about it and she said she’ll get it, she’ll go down. She called over there first. And then, then, yeah it’s ready, your medication is ready. So she asked, I told her that she worked for the CHR and all that. She went down and got it. That was =[CHR 2 Name Omitted]= the one that….(Sheldon)*

These findings suggest that CHRs were in a position that allowed them to not only support their clients’ healthy lifestyle goals but also bolster their emotional and spiritual resilience. In this way, CHRs are helping to overcome the chasm between clients and the health care system by providing both the emotional and instrumental support that the clinical team is unable to provide.

## Discussion

**Fig. 2:**
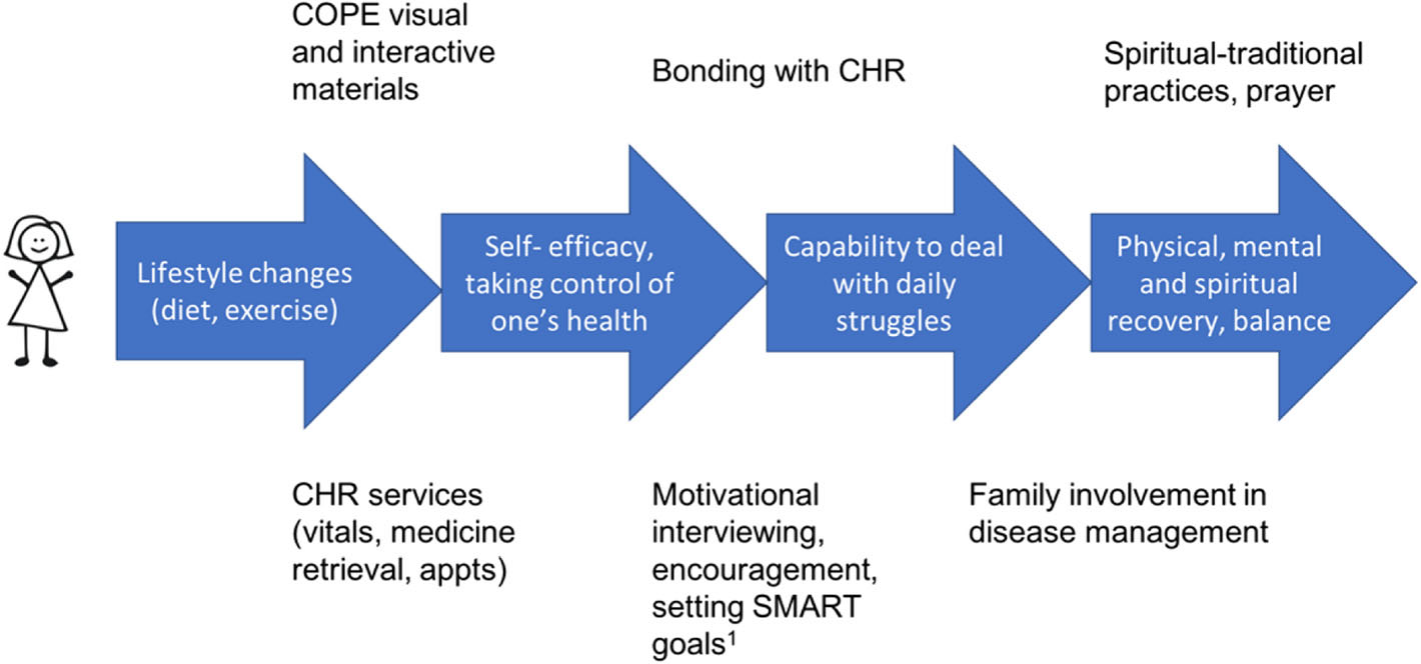
Patient Description of Path toward Wellness and Recovery. SMART: Specific, Measurable, Attainable, Relevant, Time bound

In this qualitative study, participants reported meaningful improvements in health and illness self-management. This process in its entirety is illustrated in Fig. 2.

Participants described how CHR education and coaching helped improve their ability to understand their clinical numbers and make health behavior changes. Interestingly, despite not demonstrating a full understanding of what their numbers represented, simply understanding target ranges and self-management approaches allowed participants to improve their health. We found that participants most commonly chose to focus on diet changes during the first year of COPE, and subsequently expressed growing interest in learning about physical activity.

These findings support the gradual, progressive nature of promoting health literacy and behavior change: building upon foundational concepts and small successes in behavior changes in the first year, participants are then able to further deepen their health knowledge (e.g. deepening their understanding of their numbers from simple target ranges to what the values represent) and lifestyle changes (e.g. adding physical activity while maintaining their diet changes). We have described elsewhere how clinical improvements among COPE participants have been sustained for 24 months after enrollment [19]. The findings from this study suggest that long-term clinical improvements were achieved through constant, ongoing support and feedback by the CHR and family to progressively deepen the participant’s understanding of his/her health and reinforce stepwise behavior modification to empower them toward greater self-management.

It is clear that CHRs and family members played a critical role to promote participant resilience and improved health. The unique, close-knit relationship between the CHR and their client serves as a key component in the CHR’s ability to impart knowledge of health management strategies. We have previously described the intimate link of trust between the CHR and their client [24] from the perspective of the CHR; the findings of this paper confirm that CHR clients view this relationship in a very similar way – as a vital bond that provides emotional support and a lifeline to services for individuals who are often marginalized from formal healthcare services due to limited access and mistrust. This finding is confirmed in literature that demonstrates that CHRs are more effective when they are respected by the community they serve [25].

Family members also benefitted from the educational material CHRs provided, reflecting the importance of developing interventions that target families rather than individual CHR clients. COPE and organizations aiming to improve health in Indian country through community-based interventions could benefit from formally incorporating family into care, and likewise providing additional support for these caregivers​. This may be particularly important with clients who are not readily receiving family support. CHRs could be trained to foster this bond between clients and family members or friends, and in turn, boost PROs such as successfully managing illness during times of poor health. We did not find any evidence in the literature of this being formally integrated into a community-based program.

Participants also spontaneously described another important means of self-management of health issues and life events: spirituality and traditional teachings. Participants were able to pull from traditional knowledge to create their own tailored illness management practices, which often included traditional teachings, spiritual ceremonies or rituals, artistic endeavors, and interconnectedness with family and community. These findings suggest that additional materials and CHR training could be developed that promote traditional and spiritual practices as a means to improve health outcomes and overall well-being.

These findings echo literature that suggests that CHWs worldwide are most effective when they are intimately embedded with their patient population and have materials and support they need [26], both of which we believe are the case here.

Limitations of this study stem from the difficulty in isolating components associated with the “COPE intervention” compared to the already-existing CHR program. This deeply integrated programmatic intervention was intentionally designed to layer upon existing assets to bring benefits to the CHRs and their clients in the form of a streamlined program, tailored teaching materials, and coaching methodologies focused on principles of behavior change. The study only measures change over the course of a year. It is possible that this timeframe is too short to see longer and more deeply rooted change due to the COPE intervention.

Additionally, challenges related to recruitment of study subjects hinder the generalizability of the findings. Two CHRs did not refer any clients for interviews, which could represent a bias wherein clients with positive relationships with their CHRs or experiences with the COPE intervention were more likely to participate. Thematic saturation was not achieved with this small sample size, however, given the scarcity of research evaluating programs intended to improve health outcomes for native peoples in the United States, we believe it still provides a meaningful look into health outcomes, as experienced by participants themselves.

The study team is aware that translation from Navajo to English can vary depending on the geographic location, age, and other contextual factors of the translator. Interviews with Navajo speakers were all done by the same interviewer; however, some interviews were conducted in English. This could have caused some variety in how participants understood and responded to interview questions. For this reason, in order to limit bias in translation, we carefully translated the interview guide into Navajo with the help of the interviewer and another COPE staff. We believe agreeing on the Navajo language used and soliciting open-ended answers from participants helped overcome this potential limitation. Finally, this population was a cross-section of clients seen by CHRs and receiving COPE, therefore sampling and results could be affected by contextual factors such as seasonal conditions or CHR workload.

Based on these findings, we suggest the following recommendations: 1) COPE and the Navajo Nation CHR Program could further honor participant expectations by working to deepen their understanding of clinical indicators, such as A1c and cholesterol and provide continued support for healthy food choices and portion size using visual and interactive materials as well as MI techniques; 2) More explicit incorporation of spiritual-traditional knowledge in educational materials and during CHR trainings could also help participants receive a more comprehensive and tailored experience across service units and achieve larger personal goals; 3) Finally, formally incorporating caring family members into CHR visits could improve participant health management in the home. These findings may be applicable in other native communities where other CHRs or their equivalent provide healthcare in the home. Further research may be aimed at tracking CHR changes over an extended amount of time to look at long-term PROs, compare other programs that involve CHRs or their equivalent, or that attempt to quantify other meaningful PROs through culturally appropriate surveys.

## Conclusion

Models of community outreach such as COPE and the Navajo Nation CHR Program are impactful in terms of empowering participants to improve their health and ability to understand and manage their health issues and life events. Taken together with data that demonstrates sustained improvements in clinical outcomes [19], the findings presented here support the impact on health and well-being of community-based models among individuals who are marginalized from health care services and can deeply benefit from community outreach and support.

## Supplementary information

**Additional file 1.** Qualitative Interview Questions

**Additional file 2.** Example of Interview Debrief Form

## Data Availability

All research data is property of the Navajo Nation as per Navajo Human Research Review Board (NHRRB) protocols. Investigators who seek to use this data would need to request permission from the NHRRB and provide reassurance that their request is consistent with applicable privacy, confidentiality and other legal requirements.

## Abbreviations

CAG: COPE advisory group
CHAP: Community health advisory panel
CHR: Community health representative
COPE: Community outreach and patient empowerment
IRB: Institutional review board
MI: Motivational interviewing
NHRRB: Navajo nation human research review board
PCORI: Patient-centered outcomes research institute
PRO: Patient-reported outcome
SU’s: Navajo nation service units
