# Supplementary material for: Qualitative evaluation of a community health representative program on patient experiences in Navajo Nation
Source: BMC Health Serv Res. 2020 Jan 8;20:24. doi: 10.1186/s12913-019-4839-x (PMC6950858; doi:10.1186/s12913-019-4839-x)
Supplement: Supplementary file 1 — Additional file 1. Qualitative Interview Questions [file 12913_2019_4839_MOESM1_ESM.docx]

**Interview Guide**

| Background | Where are you from? |
| --- | --- |
|  | How long have you lived here? |
|  | Who do you live with? |
|  | At which facility do you get your care? |
|  | How long have you been working with your CHR? |
| Understanding Client/Patient Reported Outcomes (PROs) | How do you feel about your overall health? (Mentally, physically, and spiritually?) |
|  | How has your health changed over the past year? |
|  | We have heard from other clients that it is important to have balance in their lives / walking in beauty. Do you feel you are able to have balance in your life? Is this something you have been able to do or has it been difficult? In what way? |
|  | Over the past year, can you tell me about a time when you were really happy with your health (or with your life in general)? How were you able to make this happen? |
|  | Now, can you tell me about the most difficult time in the past year? How did you manage to get through that situation? |
|  | Who are the people that you are closest to? Do they support you more when you’re ill? |
| Understanding of clinical numbers and outcomes | Can you tell me what you know about your health problems? (Including diagnoses. Do they understand what a A1c or BP means? Do they know their last values? Do they know which medications they take?) |
|  | How has your CHR helped you understand your health issues? |
|  | How open and comfortable do you feel about talking with your provider? Do you feel like this has gotten better or worse in the past year? Do you feel that your CHR has helped you communicate better with your provider? |
| The CHR-client relationship | Can you tell me about what it was like when the CHR first started visiting you? How did you feel about these visits? |
|  | How has your relationship changed with your CHR since then or in the past year? |
|  | Can you tell me whether you have grown to trust and confide in your CHR? |
|  | How often does your CHR visit you? Does she ever use flipcharts or handouts to teach you about health topics? How helpful are these flipcharts and handouts? How have they changed your health, if at all? |
|  | How has your CHR help you make changes? (Refer to changes referred to earlier or ask them to point out a couple examples where he/she though the CHR helped improve these outcomes?) |
|  | Are there things that you enjoy less about these visits? What do you enjoy the most about the CHR home visits? |
