# Supplementary material for: Qualitative evaluation of a community health representative program on patient experiences in Navajo Nation
Source: BMC Health Serv Res. 2020 Jan 8;20:24. doi: 10.1186/s12913-019-4839-x (PMC6950858; doi:10.1186/s12913-019-4839-x)
Supplement: Supplementary file 2 — Additional file 2. Example of Interview Debrief Form [file 12913_2019_4839_MOESM2_ESM.docx]

**Example of Debrief Form**

Date:

Time:

Interviewer Name:

Note take Name:

Interviewee Code:

Location of Interview:

Description of Interviewee

Observation of interviewee’s attitude

Describe the home environment and access to the home

Access to services (circle one)

Access to electricity yes/no

Access to plumbing system yes/no

Type of heat coal/pellet/wood

Does this person live in an area that has been affected by uranium mining?

Observations from the Interview

Were there any moment(s) during the interview that the interviewee displayed a strong emotion (positive or negative)? Describe.

Did the interview flow well? Describe any moments where the interviewee seemed uncomfortable or there was an interruption.

What were the main themes that were discussed in this interview?

1)

2)

3)

4)

Are there any emerging themes that this interview confirmed or contradicted?

What surprised you the most during the interview?

Were there any questions you would like to consider changing, adding or deleting for future interviews?

Additional observations
